# Interpretable surface-based detection of focal cortical dysplasias: a Multi-centre Epilepsy Lesion Detection study

**DOI:** 10.1093/brain/awac224

**Published:** 2022-08-10

**Authors:** Hannah Spitzer, Mathilde Ripart, Kirstie Whitaker, Felice D’Arco, Kshitij Mankad, Andrew A Chen, Antonio Napolitano, Luca De Palma, Alessandro De Benedictis, Stephen Foldes, Zachary Humphreys, Kai Zhang, Wenhan Hu, Jiajie Mo, Marcus Likeman, Shirin Davies, Christopher Güttler, Matteo Lenge, Nathan T Cohen, Yingying Tang, Shan Wang, Aswin Chari, Martin Tisdall, Nuria Bargallo, Estefanía Conde-Blanco, Jose Carlos Pariente, Saül Pascual-Diaz, Ignacio Delgado-Martínez, Carmen Pérez-Enríquez, Ilaria Lagorio, Eugenio Abela, Nandini Mullatti, Jonathan O’Muircheartaigh, Katy Vecchiato, Yawu Liu, Maria Eugenia Caligiuri, Ben Sinclair, Lucy Vivash, Anna Willard, Jothy Kandasamy, Ailsa McLellan, Drahoslav Sokol, Mira Semmelroch, Ane G Kloster, Giske Opheim, Letícia Ribeiro, Clarissa Yasuda, Camilla Rossi-Espagnet, Khalid Hamandi, Anna Tietze, Carmen Barba, Renzo Guerrini, William Davis Gaillard, Xiaozhen You, Irene Wang, Sofía González-Ortiz, Mariasavina Severino, Pasquale Striano, Domenico Tortora, Reetta Kälviäinen, Antonio Gambardella, Angelo Labate, Patricia Desmond, Elaine Lui, Terence O’Brien, Jay Shetty, Graeme Jackson, John S Duncan, Gavin P Winston, Lars H Pinborg, Fernando Cendes, Fabian J Theis, Russell T Shinohara, J Helen Cross, Torsten Baldeweg, Sophie Adler, Konrad Wagstyl

**Affiliations:** Institute of Computational Biology, Helmholtz Center Munich, Munich 85764, Germany; Department of Developmental Neuroscience, UCL Great Ormond Street Institute for Child Health, London WC1N 1EH, UK; The Alan Turing Institute, London NW1 2DB, UK; Great Ormond Street Hospital NHS Foundation Trust, London WC1N 3JH, UK; Great Ormond Street Hospital NHS Foundation Trust, London WC1N 3JH, UK; Penn Statistics in Imaging and Visualization Center, Department of Biostatistics, Epidemiology, and Informatics, University of Pennsylvania, Philadelphia, PA 19104, USA; Center for Biomedical Image Computing and Analytics, University of Pennsylvania, Philadelphia, PA 19104, USA; Medical Physics Department, Bambino Gesù Children’s Hospital, Rome 00165, Italy; Rare and Complex Epilepsies, Department of Neurosciences, Bambino Gesù Children’s Hospital, IRCCS, Rome 00165, Italy; Neurosurgery Unit, Department of Neurosciences, Bambino Gesù Children’s Hospital, IRCCS, Rome 00165, Italy; Barrow Neurological Institute at Phoenix Children’s Hospital, Phoenix, AZ 85016, USA; Barrow Neurological Institute at Phoenix Children’s Hospital, Phoenix, AZ 85016, USA; Department of Neurosurgery, Beijing Tiantan Hospital, Capital Medical University, Beijing 100054, China; Department of Neurosurgery, Beijing Tiantan Hospital, Capital Medical University, Beijing 100054, China; Department of Neurosurgery, Beijing Tiantan Hospital, Capital Medical University, Beijing 100054, China; Bristol Royal Hospital for Children, Bristol BS2 8BJ, UK; School of Psychology, Cardiff University Brain Research Imaging Centre, Cardiff CF24 4HQ, UK; The Welsh Epilepsy Unit, Cardiff and Vale University Health Board, University Hospital of Wales, Cardiff CF14 4XW, UK; Charité University Hospital, Berlin 10117, Germany; Neuroscience Department, Children’s Hospital Meyer-University of Florence, Florence 50139, Italy; Center for Neuroscience, Children’s National Hospital, Washington, DC 20012, USA; Department of Neurology, West China Hospital of Sichuan University, Chengdu 610093, China; Epilepsy Center, Cleveland Clinic, Cleveland, OH 44106, USA; Epilepsy Center, Cleveland Clinic, Cleveland, OH 44106, USA; Department of Neurology, Epilepsy Center, Second Affiliated Hospital, School of Medicine, Zhejiang University, Hangzhou 310058, China; Department of Developmental Neuroscience, UCL Great Ormond Street Institute for Child Health, London WC1N 1EH, UK; Great Ormond Street Hospital NHS Foundation Trust, London WC1N 3JH, UK; Department of Developmental Neuroscience, UCL Great Ormond Street Institute for Child Health, London WC1N 1EH, UK; Great Ormond Street Hospital NHS Foundation Trust, London WC1N 3JH, UK; Department of Neuroradiology, Hospital Clinic Barcelona and Magnetic Resonance Imaging, Core Facility, IDIBAPS, Barcelona 08036, Spain; Centro de Investigación Biomédica en Red de Salud Mental, CIBERSAM, Madrid 28029, Spain; Magnetic Resonance Imaging, Core Facility, IDIBAPS, Barcelona 08036, Spain; Magnetic Resonance Imaging, Core Facility, IDIBAPS, Barcelona 08036, Spain; Magnetic Resonance Imaging, Core Facility, IDIBAPS, Barcelona 08036, Spain; Department of Neurosurgery, Hospital del Mar, Barcelona 08003, Spain; Department of Neurology, Hospital del Mar, Barcelona 08003, Spain; IRCCS Istituto Giannina Gaslini, Genova 16147, Italy; Center for Neuropsychiatry and Intellectual Disability, Psychiatrische Dienste Aargau AG, Windisch 5120, Switzerland; Institute of Psychiatry, Psychology and Neuroscience, King’s College, London SE5 8AF, UK; Institute of Psychiatry, Psychology and Neuroscience, King’s College, London SE5 8AF, UK; Department of Perinatal Imaging and Health, St. Thomas’ Hospital, King’s College London, London SE1 7EH, UK; Department of Perinatal Imaging and Health, St. Thomas’ Hospital, King’s College London, London SE1 7EH, UK; Department of Forensic and Neurodevelopmental Sciences, Institute of Psychiatry, Psychology and Neuroscience, King’s College, London SE5 8AF, UK; Department of Neurology, University of Eastern Finland, Kuopio 70210, Finland; Department of Medical and Surgical Sciences, Magna Graecia University of Catanzaro, Catanzaro 88100, Italy; Department of Neuroscience, Central Clinical School, Monash University, Melbourne, VIC 3004, Australia; Department of Neuroscience, Central Clinical School, Monash University, Melbourne, VIC 3004, Australia; Department of Neurology, Monash University, Melbourne, VIC 3004, Australia; Department of Neuroscience, Central Clinical School, Monash University, Melbourne, VIC 3004, Australia; Royal Hospital for Children and Young People, Edinburgh EH16 4TJ, UK; Royal Hospital for Children and Young People, Edinburgh EH16 4TJ, UK; Royal Hospital for Children and Young People, Edinburgh EH16 4TJ, UK; The Florey Institute of Neuroscience and Mental Health, University of Melbourne, Parkville, VIC 3052, Australia; Neurobiology Research Unit, Copenhagen University Hospital—Rigshospitalet, Copenhagen 2100, Denmark; Neurobiology Research Unit, Copenhagen University Hospital—Rigshospitalet, Copenhagen 2100, Denmark; Department of Neuroradiology, Copenhagen University Hospital—Rigshospitalet, Copenhagen 2100, Denmark; Department of Neurology, University of Campinas, Campinas 13083-888, Brazil; Brazilian Institute of Neuroscience and Neurotechnology (BRAINN), University of Campinas, Campinas 13083-888, Brazil; Department of Neurology, University of Campinas, Campinas 13083-888, Brazil; Brazilian Institute of Neuroscience and Neurotechnology (BRAINN), University of Campinas, Campinas 13083-888, Brazil; Neuroradiology Unit, IRCCS Bambino Gesù Children’s Hospital, Rome 00165, Italy; School of Psychology, Cardiff University Brain Research Imaging Centre, Cardiff CF24 4HQ, UK; The Welsh Epilepsy Unit, University Hospital of Wales, Cardiff CF14 4XW, UK; Charité University Hospital, Berlin 10117, Germany; Neuroscience Department, Children’s Hospital Meyer-University of Florence, Florence 50139, Italy; Neuroscience Department, Children’s Hospital Meyer-University of Florence, Florence 50139, Italy; Center for Neuroscience, Children’s National Hospital, Washington, DC 20012, USA; Center for Neuroscience, Children’s National Hospital, Washington, DC 20012, USA; Epilepsy Center, Cleveland Clinic, Cleveland, OH 44106, USA; Department of Neuroradiology, Hospital del Mar, Barcelona 08003, Spain; Magnetic Resonance Imaging Core Facility, Institut d’Investigacions Biomèdiques August Pi i Sunyer (IDIBAPS), Barcelona 08036, Spain; IRCCS Istituto Giannina Gaslini, Genova 16147, Italy; IRCCS Istituto Giannina Gaslini, Genova 16147, Italy; Department of Neurosciences, Rehabilitation, Ophthalmology, Genetics, Maternal and Child Health, University of Genova, Genova, Italy; IRCCS Istituto Giannina Gaslini, Genova 16147, Italy; Department of Neurology, University of Eastern Finland, Kuopio 70210, Finland; Kuopio Epilepsy Center, Neurocenter, Kuopio University Hospital, Kuopio 70210, Finland; Institute of Neurology, Department of Medical and Surgical Sciences, Magna Graecia University, Catanzaro 88100, Italy; Neurology Unit, Department of BIOMORF, University of Messina, Messina 98168, Italy; Department of Radiology, The Royal Melbourne Hospital, University of Melbourne, Parkville, VIC 3050, Australia; Department of Radiology, The Royal Melbourne Hospital, University of Melbourne, Parkville, VIC 3050, Australia; Department of Neuroscience, Central Clinical School, Monash University, Melbourne, VIC 3004, Australia; Department of Medicine, The Royal Melbourne Hospital, Parkville, VIC, 3052, Australia; Royal Hospital for Children and Young People, Edinburgh EH16 4TJ, UK; The Florey Institute of Neuroscience and Mental Health, Austin Campus, Heidelberg, VIC 3071, Australia; Department of Neurology, Austin Health, Heidelberg, VIC 3084, Australia; UCL Queen Square Institute of Neurology, London WC1N 3BG, UK; UCL Queen Square Institute of Neurology, London WC1N 3BG, UK; Department of Medicine, Division of Neurology, Queen’s University, Kingston, ON, Canada K7L 3N6; Neurobiology Research Unit, Copenhagen University Hospital—Rigshospitalet, Copenhagen 2100, Denmark; Epilepsy Clinic, Department of Neurology, Copenhagen University Hospital—Rigshopsitalet, Copenhagen 2100, Denmark; Department of Neurology, University of Campinas, Campinas 13083-888, Brazil; Brazilian Institute of Neuroscience and Neurotechnology (BRAINN), University of Campinas, Campinas 13083-888, Brazil; Institute of Computational Biology, Helmholtz Center Munich, Munich 85764, Germany; Department of Mathematics, Technical University of Munich, Garching 85748, Germany; Penn Statistics in Imaging and Visualization Center, Department of Biostatistics, Epidemiology, and Informatics, Perelman School of Medicine, University of Pennsylvania, Philadelphia, PA 19104, USA; Department of Developmental Neuroscience, UCL Great Ormond Street Institute for Child Health, London WC1N 1EH, UK; Young Epilepsy, Lingfield, Surrey RH7 6PW, UK; Department of Developmental Neuroscience, UCL Great Ormond Street Institute for Child Health, London WC1N 1EH, UK; Great Ormond Street Hospital NHS Foundation Trust, London WC1N 3JH, UK; Department of Developmental Neuroscience, UCL Great Ormond Street Institute for Child Health, London WC1N 1EH, UK; Department of Developmental Neuroscience, UCL Great Ormond Street Institute for Child Health, London WC1N 1EH, UK; Wellcome Centre for Human Neuroimaging, University College London, London WC1N 3AR, UK

**Keywords:** focal cortical dysplasia, epilepsy, structural MRI, machine learning

## Abstract

One outstanding challenge for machine learning in diagnostic biomedical imaging is algorithm interpretability. A key application is the identification of subtle epileptogenic focal cortical dysplasias (FCDs) from structural MRI. FCDs are difficult to visualize on structural MRI but are often amenable to surgical resection. We aimed to develop an open-source, interpretable, surface-based machine-learning algorithm to automatically identify FCDs on heterogeneous structural MRI data from epilepsy surgery centres worldwide.

The Multi-centre Epilepsy Lesion Detection (MELD) Project collated and harmonized a retrospective MRI cohort of 1015 participants, 618 patients with focal FCD-related epilepsy and 397 controls, from 22 epilepsy centres worldwide. We created a neural network for FCD detection based on 33 surface-based features. The network was trained and cross-validated on 50% of the total cohort and tested on the remaining 50% as well as on 2 independent test sites. Multidimensional feature analysis and integrated gradient saliencies were used to interrogate network performance.

Our pipeline outputs individual patient reports, which identify the location of predicted lesions, alongside their imaging features and relative saliency to the classifier. On a restricted ‘gold-standard’ subcohort of seizure-free patients with FCD type IIB who had T_1_ and fluid-attenuated inversion recovery MRI data, the MELD FCD surface-based algorithm had a sensitivity of 85%. Across the entire withheld test cohort the sensitivity was 59% and specificity was 54%. After including a border zone around lesions, to account for uncertainty around the borders of manually delineated lesion masks, the sensitivity was 67%.

This multicentre, multinational study with open access protocols and code has developed a robust and interpretable machine-learning algorithm for automated detection of focal cortical dysplasias, giving physicians greater confidence in the identification of subtle MRI lesions in individuals with epilepsy.

## Teaser

Spitzer *et al.* present a robust and interpretable open-source machine-learning algorithm for automated detection of focal cortical dysplasias. The pipeline outputs individual patient reports, which identify the location of predicted lesions, alongside their imaging features and relative saliency to the classifier.

## Introduction

The application of machine learning algorithms for diagnostics in biomedical imaging forms a spectrum from automating high-throughput imaging analysis to assisting diagnosis in rarer, clinically challenging pathologies. One barrier to clinical translation is the limited interpretability of these algorithms, leading to a common perception of them as impenetrable ‘black boxes’. Identifying focal epileptogenic abnormalities on MRI is an outstanding clinical challenge in patients undergoing presurgical evaluation for drug-resistant focal epilepsy (DRFE). In DRFE, 16–43% of individuals are ‘MRI-negative’, i.e. no relevant abnormality is visually identified on their MRI scans.^1–3^ A leading cause of DRFE and the most common histopathology in operated ‘MRI-negative’ cohorts is a malformation of cortical development, called focal cortical dysplasia (FCD).^4^ As post-surgical seizure freedom is affected by whether the FCD can be identified on preoperative structural MRI,^1,5^ there has been considerable effort placed in improving the detection of these lesions. However, machine-learning approaches provide little insight into factors determining classification. In clinically ambiguous images, where the need for algorithms is greatest, such insight would enable physicians to determine whether features identified by the classifiers are likely to be lesional in origin.

Radiologically, FCDs are characterized by alterations in cortical thickness, blurring at the grey–white matter boundary, folding abnormalities and T_2_ or fluid-attenuated inversion recovery (FLAIR) signal intensity changes.^3^ Approaches to improving the detection of FCDs have involved improved scanner protocols^6^ and field strengths^7,8^ as well as automated volumetric-^9–13^ and surface-based^14–17^ post-processing methods.

Despite extensive retrospective work to improve FCD detection, few automated methods have been used prospectively in the presurgical evaluation of patients with epilepsy. Alongside lack of interpretability, there are many additional reasons for this. Initially, many of the frameworks were developed at single epilepsy centres, resulting in small sample sizes and homogeneous datasets, where all patients have been scanned on the same MRI scanner with the same protocol, which reduces the likelihood of robustness of the results and the ability of the method to generalize. Many of these frameworks are not openly available and therefore difficult to reproduce. Although there has been some important research replicating previous methods,^15,18,19^ there was a need to develop and validate automated FCD detection tools on multicentre data. Recently, the field has progressed with two large multicentre studies,^11,12^ which successfully trained neural networks on voxel-based MRI data from 13 and 11 MRI scanners, respectively, to detect FCDs. However, neither of these studies included any patients with FCD type I lesions, which are particularly difficult to diagnose and represent some of the complex, challenging patients who present to epilepsy surgery centres.

Here, as part of the Multi-centre Epilepsy Lesion Detection (MELD) Project,^20^ we aimed to collate a heterogeneous cohort of patients from multiple epilepsy surgery centres, across multiple MRI scanners including both 1.5 and 3 T field strengths; create protocols for decentralized MRI post-processing; and develop an open-access, robust and interpretable surface-based classifier to detect FCD.

## Materials and methods

### MELD project consortium

The MELD project (https://meldproject.github.io/) involves 22 research centres across 5 continents. Each centre received approval from their local institutional review board (IRB) or ethics committee (EC). IRB/EC waived the need for individual patient consent as this was a retrospective study using fully anonymized, routinely available data only.

### Participants

Patients were included if they were over age 3, had a 3D preoperative T_1_-weighted MRI brain scan (1.5 or 3 T) and a radiological diagnosis of FCD or were MRI-negative with histopathological confirmation of FCD. Participants were excluded if they had previous surgery, large structural abnormalities in addition to the FCD or T_1_ scans with gadolinium enhancement. Controls were included if they were over age 3, did not have epilepsy or another neurological condition and had a T_1_-weighted MRI brain scan (1.5 or 3 T). Patients scanned for headache could be included as controls if they had no other neurological conditions and the MRI was normal. The patients and controls included were a retrospective convenience sample. Centres, patients and controls were given pseudo-anonymized ID codes.

### Methods overview

**Figure 1:**
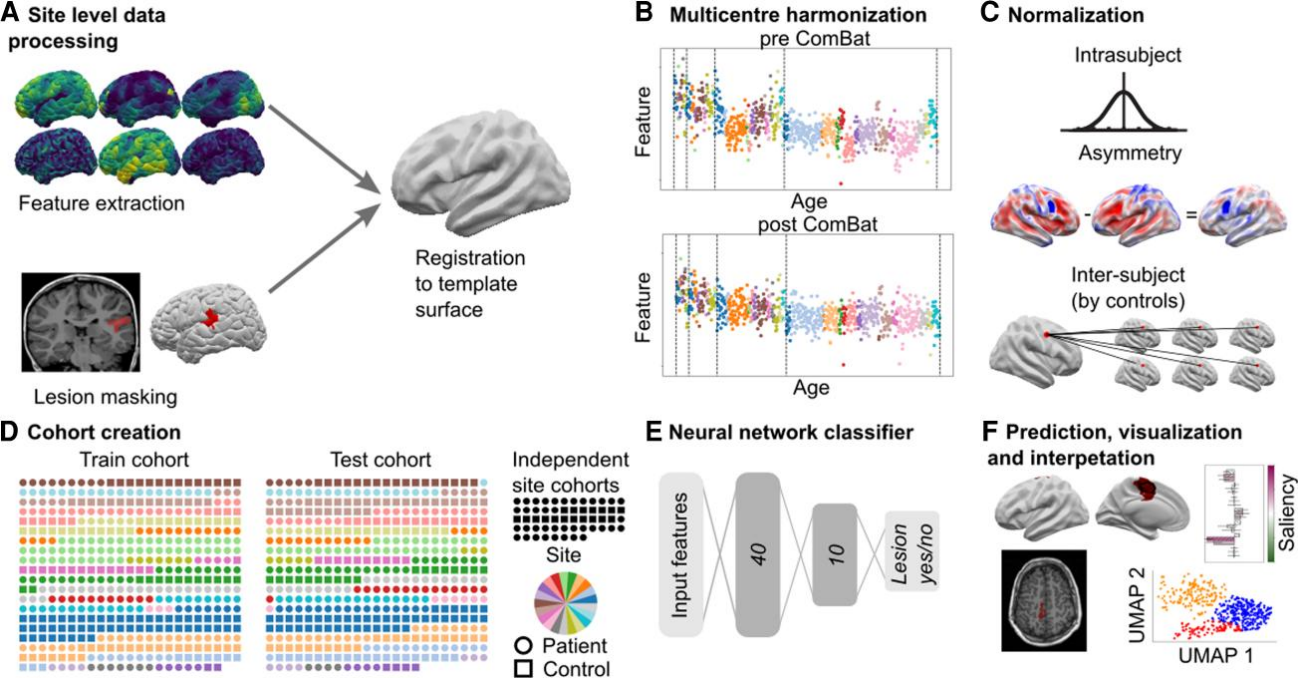
**MELD processing pipeline.** (**A**) Local sites extract surface-based morphological features from structural T_1_ and FLAIR MRI, along with manually delineated lesion masks. These were coregistered to a symmetric template surface and anonymized data matrices are shared with the MELD team. (**B**) Central preprocessing: the MELD team carried out outlier detection and data harmonization to minimize interscanner feature differences. (**C**) Morphological features underwent intrasubject, interhemispheric and intersubject normalization. (**D**) The full cohort was randomly subdivided 50:50 into training/validation cohorts and withheld test cohort. To avoid overfitting, all optimization experiments were carried out on the training/validation cohort prior to final testing on the test cohort and new site cohorts. (**E**) The neural network classifier was trained to identify lesional vertices from MRI features. Vertex-wise predictions were collected into connected clusters. (**F**) Classifier predictions mapped to cortical surfaces, lesional features and their relative saliency were plotted; lesional features across the cohort were analysed.

Fig. 1 is an overview of the MELD FCD processing pipeline, which is explained in more detail in the sections below.

### Site-level data collection and post-processing

Each site followed the protocols for site-level data collection and post-processing that are available at https://www.protocols.io/researchers/meld-project and detailed in the following sections ‘Participant demographics’, ‘MRI data collection and cortical surface reconstruction’, ‘FCD lesion masking’ and ‘Morphological/intensity features’. Structural MRI post-processing protocols were adapted from openly available ENIGMA-epilepsy protocols.^21^

#### Participant demographics

The following data were collected for all patients: age at preoperative scan, sex, age of epilepsy onset, duration of epilepsy (time from age of epilepsy onset to age at preoperative scan), ever reported MRI-negative and histopathological diagnosis (ILAE three-tiered classification system),^22^ seizure freedom (Engel class I or other) and follow-up time in operated patients.

#### MRI data collection and cortical surface reconstruction

3D T_1_-weighted and FLAIR (where available) MRI scans were collected at the 22 participating centres for all participants. We included MRI data acquired on Siemens, GE and Philips MRI scanners at either 1.5 or 3 T field strengths. Cortical surfaces were reconstructed using *FreeSurfer*.^23^ Sites could process their data using either Linux or Mac operating systems and use either *FreeSurfer* v5.3 or v6.

#### FCD lesion masking

FCD lesions were delineated on the T_1_-weighted MRI scans at each site according to our lesion masking protocol.^24^ For patients with a radiological diagnosis of FCD, a volumetric lesion mask was created using the preoperative T_1_ scan and 3D FLAIR (where available). For MRI-negative patients but with histopathological confirmation of FCD, the postoperative scan was used to identify the location of the FCD on the preoperative T_1_ or FLAIR. A volumetric lesion mask was then created on the preoperative MRI data. In both cases, masks were created by a neuroradiologist, neurologist or experienced epilepsy researcher at each site. Volumetric lesion masks were mapped to cortical reconstructions and small defects were filled in using five iterations of a dilation–erosion algorithm. Patients’ lesions were registered to *fsaverage_sym*.

Interrater reliability in lesion masking was assessed by three expert neuroradiologists independently masking on 10 randomly chosen FCD lesions from one site.

#### Morphological/intensity features

The following measures were calculated in native space per vertex across the cortical surface in all participants: (i) cortical thickness; (ii) grey–white contrast; (iii) mean curvature; (iv) sulcal depth; and (v) intrinsic curvature. Thickness was calculated as the mean minimum distance (in millimetres) between each vertex on the pial and white matter surfaces.^25^ Grey–white contrast was calculated as the ratio of the T_1_ grey matter signal intensity (at 30% of the cortical thickness) to the white matter signal intensity (1 mm below the grey–white matter boundary).^26^ Mean curvature was calculated at the grey–white matter boundary as 1/*r*, where *r* is equal to the mean of the principal curvatures *k*1 and *k*2.^27^ The dot product of the movement vector of the cortical surface during inflation is used to calculate the sulcal depth. Intrinsic curvature was calculated as the dot product of the principal curvatures *k*1 and *k*2.^28^

In participants with FLAIR data, FLAIR signal intensity was sampled at 25%, 50%, and 75% of the cortical thickness (GM FLAIR 25%, 50%, 75%), as well as at the grey–white matter boundary and 0.5 and 1 mm subcortically (WM FLAIR 0.5 mm, 1 mm).

To increase the stability of per-vertex measures, the following features were smoothed with a 5 mm Gaussian kernel: mean curvature and sulcal depth; and 10 mm Gaussian kernel: cortical thickness, grey–white contrast and FLAIR intensities at all cortical and subcortical depths. Intrinsic curvature was smoothed with a 20 mm Gaussian kernel to provide a measure of folding pattern abnormalities that is stable across adjacent gyri and sulci. All features were registered to bilaterally symmetrical template space, *fsaverage_sym*. Only anonymized participant demographic details and data matrices of anonymized features and lesion masks were shared with the MELD Project coordinators for multicentre analysis.

### Centralized quality control and post-processing

#### Quality control and data harmonization of surface-based data

Automated quality control was performed on the surface-based features to identify subjects with extreme structural and intensity values across multiple features and cortical areas, likely caused by imaging artefacts such as signal biases or *FreeSurfer* segmentation errors. A feature was considered an outlier if, in more than 10 non-lesional regions (from the Desikan–Killiany atlas), it was greater or less than 2.7 times the standard deviation from the mean of all participants’ values.^21^ Participants were considered outliers if they had multiple extreme features, two if features from T_1_-weighted scans only and three if FLAIR MRI scans available. Participants identified as outliers were excluded from all subsequent analyses. For further details see Supplementary Fig. 1.

Due to heterogeneity in MRI scanner hardware, scanner field strength, operating systems and *FreeSurfer* versions, which can all affect morphological and intensity feature values,^29^ features were harmonized using ComBat^30^ to control for non-biological variance while retaining biological covariates (age, sex and disease status; Supplementary Fig. 2). Independent test sites were harmonized to the main cohort (Supplementary Fig. 2B). The harmonized data set features are henceforth referred to as ‘ComBat’ features.

#### Three-stage normalization of features

Surface-based MRI features underwent three normalization procedures to highlight feature abnormalities.

Step 1: To account for interindividual shifts in feature distributions, such as age and sex-related changes, features were normalized using intrasubject *z-*scoring. For example, the cortex is thicker in a 3-year-old than in a 60-year-old (Supplementary Fig. 2A). After intrasubject *z*-scoring, thickness metrics for both participants will all have a mean of 0 and a standard deviation of 1.

To account for interregional variability in features, two further normalization steps were carried out: interhemispheric asymmetry and per-vertex normalization by controls.

Step 2: Interhemispheric asymmetry maps of features were created by subtracting right hemisphere vertex values from left hemisphere values and vice versa. This procedure leverages the normal symmetry of cortical morphometric features and quantifies a key heuristic used to detect FCDs on radiological review, highlighting vertices that are significantly different from the contralateral side.

Step 3: The outputs from steps 1 and 2 were *z*-scored by the mean and standard deviation of features at each vertex from healthy controls to adjust for normal interregional variability. For example, the cortex in frontal regions is normally thicker than in the occipital cortex. By normalizing by the control values at each vertex, we can account for this normal variability to accentuate features that are abnormal for their position in the cortex.

The output of these normalization steps is a set of intrasubject and intersubject normalized features (henceforth ‘normalized’ features) and a set of intrasubject, asymmetry and intersubject normalized featured (henceforth ‘asymmetry’ features).

#### Characterization of focal cortical dysplasia features on MRI

Surface-based morphological features were calculated within the lesion masks of all patients. For controls, data were sampled from similarly sized regions for comparison. T_1_-derived features, available in all subjects, underwent Uniform Manifold Approximation and Projection (UMAP) embedding,^31^ a non-linear dimensionality reduction where similar examples are plotted closer together. Lesions were clustered into groups according to their UMAP locations using a Gaussian mixture model.

#### Border zones

Lesion masks were drawn conservatively, to maximize the proportion of lesional vertices within the mask. There is inherent uncertainty in the precise borders of manually delineated lesion masks. Feature abnormalities extended approximately 40 mm beyond the lesion (Supplementary Fig. 3). To account for this uncertainty, border zones were created around each lesion mask extending 20 and 40 mm across the cortical surface. Vertices between 0 and 40 mm from the lesion mask were excluded from training to reduce training on mislabelled data. Predicted lesion clusters within 20 mm of the lesion masks classified as detected for the sensitivity+ metric (see network evaluation section).

### Network training, testing and interpretation

#### Cohort splitting

An artificial neural network was trained on per-vertex post-processed MRI features (ComBat, Asymmetry and Normalized), after border zones had been removed (33 total input features). The full cohort (excluding two independent test sites) of patients and controls were randomly assigned to either the train cohort (278 patients, 180 controls) or the test cohort (260 patients, 193 controls) (Table 1). All experiments to determine the optimal data processing and network parameters were carried out through 10-fold cross-validation on the train cohort. The 10 folds were determined by a random partition of subjects in the train cohort. Hyperparameters were selected according to the aggregated performance metrics of each of the 10 cross-validation models on their respective validation set.

**Table 1:** Demographic Information

	Train cohort		Test cohort		Independent site 1		Independent site 2
	Patients (*n* = 278)	Controls (*n* = 180)	Patients (*n* = 260)	Controls (*n* = 193)	Patients (*n* = 17)	Controls (*n* = 18)	Patients (*n* = 16)
Age at preoperative scan [median (IQR)]	20.0 (11.0–32.8)	29.0 (19.0–37.9)	18.0 (11.0–29.0)	29.0 (19.5–39.2)	7.3 (5.2–11.1)	14.6 (10.5–16.1)	6.1 (3.4–16.2)
Sex (female:male)	150:127	105:75	125:135	104:88	7:10	10:8	6:10
Age of epilepsy onset [median (IQR)]	6.0 (2.5–12.0)		6.0 (3.0–11.0)		3.4 (0.8–5.8)		2.0 (0.9–5.1)
Duration of epilepsy [median (IQR)]	10.0 (4.3–18.4)		10.2 (5.0–18.2)		3.0 (0.5–7.2)		2.4 (1.3–8.1)
FLAIR available	132/278 (47.0%)	28/180 (16.0%)	110/260 (42.0%)	28/193 (15.0%)	17/17 (100.0%)	18/18 (100.0%)	16/16 (100.0%)
Scanner (1.5:3 T)	41:237	18:162	56:204	15:178	0:17	0:17	0:16
Surgery	208/278 (75.0%)		190/260 (73.0%)		5/17 (29.0%)		16/16 (100.0%)
Histology	193/208 (93.0%)		171/190 (90.0%)		4/5 (80.0%)		16/16 (100.0%)
Seizure-free	123/183 (67.0%)		106/157 (68.0%)		3/5 (60.0%)		14/16 (88.0%)
Follow-up time	2.0 (1.0–3.0)		2.0 (1.0–3.4)		1.5 (1.1–1.7)		2.9 (1.9–4.4)

**Table 2:** Classifier performance

	Sensitivity+ (percentage of patients detected)	Sensitivity (percentage of patients detected)	Number of clusters in patients [median (IQR)]	Specificity (percentage of controls with zero clusters)	Number of clusters in controls [median (IQR)]
Test cohort	67% (174/260)	59% (154/260)	2 (1.0–3.0)	54% (105/193)	0 (0.0–1.0)
Full cohort	65% (350/538)	58% (314/538)	2 (1.0–3.0)	52% (194/373)	0 (0.0–1.0)
Independent site 1	94% (16/17)	88% (15/17)	2 (2.0–4.0)	17% (3/18)	1 (1.0–2.0)
Independent site 2	62% (10/16)	56% (9/16)	2 (2.0–3.25)	NA	NA

Performance of the classifier on the test cohort, full cohort and the two independent sites.

#### Network hyperparameters and training

The neural network architecture had two hidden layers (with 40 and 10 nodes, respectively) and one output node and used a dropout of 0.4 on the input layer for learning more robust representations. To adjust for the class imbalance between healthy and lesional examples, for each patient 2000 random lesional and non-lesional vertices were sampled per epoch. If a patient had less than 2000 lesional vertices, existing lesional vertices were randomly drawn multiple times. A focal loss^32^ was used to concentrate network training on difficult examples. After training, the network predictions were thresholded using an optimal threshold determined based on the Dice (F1) score on the train cohort. For the full list of optimized parameters see Supplementary Table 1.

The following experiments were conducted to evaluate the impact of smoothing kernel size and feature normalization on classifier performance: (i) morphological and intensity features were smoothed with Gaussian kernels ranging from 3 to 25 mm and models were retrained using these smoothed features; and (ii) three models were retrained using (a) ComBat, (b) ComBat and normalized and (c) ComBat, normalized and asymmetry features. For these experiments, analyses were restricted to the train cohort. On each of 10 folds, a classifier was trained 10 times with random initializations and an ensemble of the 10 models was evaluated on the fold’s validation cohort. Results were aggregated across the 10 folds.

For the final training and testing of the model after data and hyperparameter optimization, a classifier was trained five times with different random initializations on each of 10 training folds. The resulting 50 models were combined into one final ensemble model^33,34^ by averaging the individual models’ predictions. For every input, the final model will therefore run each of the 50 individual models and output the average lesional probability predicted by these models to increase predictive performance and stability. This final model was evaluated on the test cohort. To calculate individual performance statistics for subjects in the train cohort, a second ensemble network was trained in a similar manner on the test cohort and evaluated on the train cohort.

#### Evaluation metrics

Per-vertex lesion predictions for each individual were grouped into spatially connected clusters on the surface mesh. Clusters smaller than 100 vertices (approximately 0.5 cm^2^) were filtered out as these are disproportionately false positives (Supplementary Fig. 4). The following outcome measures were calculated: (i) sensitivity, defined as the proportion of patients where a predicted lesion cluster overlapped the manual lesion mask; (ii) sensitivity+, defined as the proportion of patients where a predicted lesion cluster overlapped the manual lesion mask or the border zone; (iii) specificity, defined as the proportion of controls with zero clusters; (iv) average number of clusters per patient; and (v) average number of clusters per control.

#### Network performance evaluation

Three complementary methods to understand and interrogate classifier performance and behaviour were used.

To determine how demographic and clinical factors influenced whether lesions were successfully detected by the classifier, two logistic regression models were used. The first included presurgically available variables: sex, scanner field strength, lesion hemisphere, FLAIR availability. The second included post-surgical variables (histopathological diagnosis and seizure freedom) and was applied on the cohort of patients who had undergone surgery. Statistical significance was determined through repeating regression analysis on randomly permuted cohorts (1000 permutations). Correction for multiple comparisons used the Benjamini–Hochberg procedure.^35^

To understand classifier predictions, MRI features from predicted clusters were transformed into the UMAP embedded space described above.

To understand which specific features drove network predictions, integrated gradients saliency was computed.^36^ This method computes which features are important to the network by looking at the integral (Riemann approximation) of the gradients computed from a baseline input (0 for each feature) to the actual feature values for each vertex.

### Data availability

All data analysis was performed in Python. All protocols and code are available to download from https://www.protocols.io/researchers/meld-project and www.github.com/MELDProject/meld_classifier. Requests for access to the MELD dataset can be made through the project website https://meldproject.github.io//.

## Results

### Participant demographics

After excluding patients with missing lesion labels (*n* = 37) and outliers (*n* = 14), a total of 571 FCD patients were included (Table 1). Each epilepsy surgery centre contributed 6–87 patients. Four hundred and nineteen patients underwent surgical intervention (73%) and histopathological diagnosis was available in 384 patients (92% of operated patients). Post-surgical outcome data were available in 361 patients (86% of operated patients); 68% were seizure free (Engel class 1) at last follow-up (median follow up = 2 years).

### Interrater agreement in lesion masking

A set of three expert-defined lesion masks were created for 10 randomly selected subjects from one site (Supplementary Fig. 5). The mean fraction mask overlap between rater–rater pairs was 42%, indicating that lesion annotations are likely to be heterogeneous. However, adding a border zone of 20 and 40 mm to the first rater’s mask led to the overlap increasing to 82% and 94%, respectively. In a binary test of whether masks overlapped, with a border zone of 20 mm, there was at least one vertex overlap between all pairs of masks.

### Focal cortical dysplasia lesion characterization

**Figure 2:**
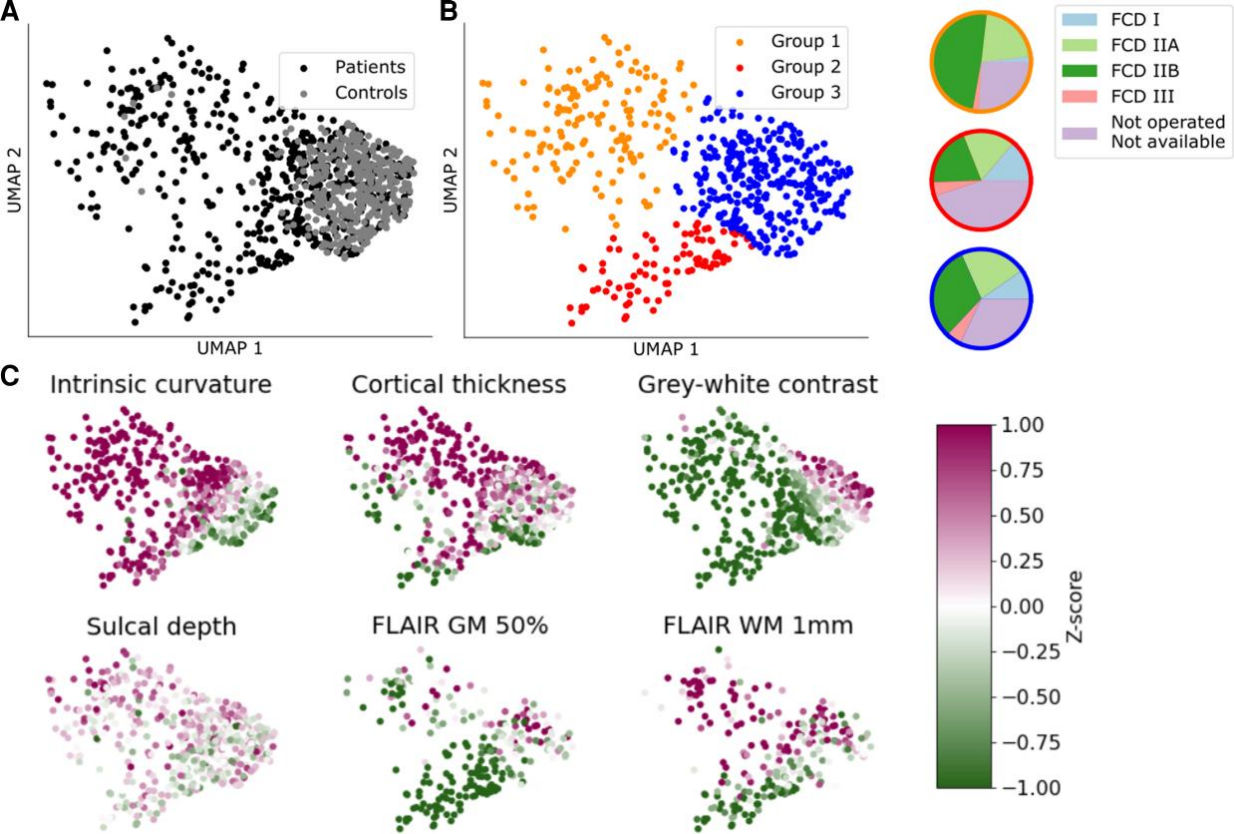
**Non-linear 2D UMAP embedding of lesional T_1_ features.** (**A**) Manual lesion masks of patients (black) compared to equivalent cortex on healthy controls (grey). Lesions differ from control cortex and exhibit different patterns of structural abnormality. (**B**) Data-driven clustering of UMAP embedding reveals three distinct groups of lesions. Colour-associated pie charts describe the proportion of each histopathological subtype present in each group. (**C**) Patient lesions coloured by intra- and intersubject normalized features. Group 1 is predominantly FCD IIA and IIB, along with unoperated patients. It is characterized by increased intrinsic curvature, increased cortical thickness, decreased grey–white matter contrast, bottom of sulcus and increased FLAIR in the white matter. Group 2 is characterized by increased intrinsic curvature, decreased grey–white matter contrast and decreased intracortical FLAIR. It contains proportionally more FCD I and III lesions. Group 3 largely overlaps healthy control clusters. Lesional features in this cluster are more heterogeneous and less extreme.

UMAP embedding of surface-based features from manual lesion masks and equivalent healthy cortex in the full cohort is shown in Fig. 2A. Compared to healthy control cortex, many lesions exhibited a distinct set of MRI features. There was heterogeneity in the set of abnormal features, with three distinct groups emerging (Fig. 2B). Group 1 was predominantly composed of FCD type IIA, IIB and unoperated lesions. These lesions were generally located at the bottom of a sulcus and characterized by increased intrinsic curvature, increased cortical thickness, decreased grey–white matter contrast and increased FLAIR in the white matter. Group 2 lesions were characterised by increased intrinsic curvature, decreased grey–white matter contrast and decreased intracortical FLAIR. Group 3 lesions, in which the lesional features overlapped with healthy cortex, were more heterogeneous and had less extreme feature values.

### Classifier performance

#### Impact of feature preprocessing on classifier performance

Performance of the classifier on the test cohort, full cohort and two independent sites are listed in Table 2. For the 278 patients in the train cohort, we assessed the impact of feature normalization procedures and smoothing kernels on classifier performance to establish the optimal input data for the classifier. There is an improvement in sensitivity+ (from 54% to 65%), sensitivity (from 44% to 59%) and in specificity (from 17% to 44%) following the three-stage normalization of the data (Supplementary Table 2). As Gaussian smoothing kernel size increased in size (Supplementary Fig. 6), classifier sensitivity decreased. However, the number of detected clusters in patients and controls also decreased (Supplementary Fig. 6). Based on these experiments we decided that using a 5 mm Gaussian kernel for sulcal depth and mean curvature, 10 mm for cortical thickness, grey–white contrast and FLAIR intensities at all cortical and subcortical depths and 20 mm for intrinsic curvature represents an acceptable trade-off between falling sensitivity and rising specificity.

#### Detection in the test cohort

**Figure 3:**
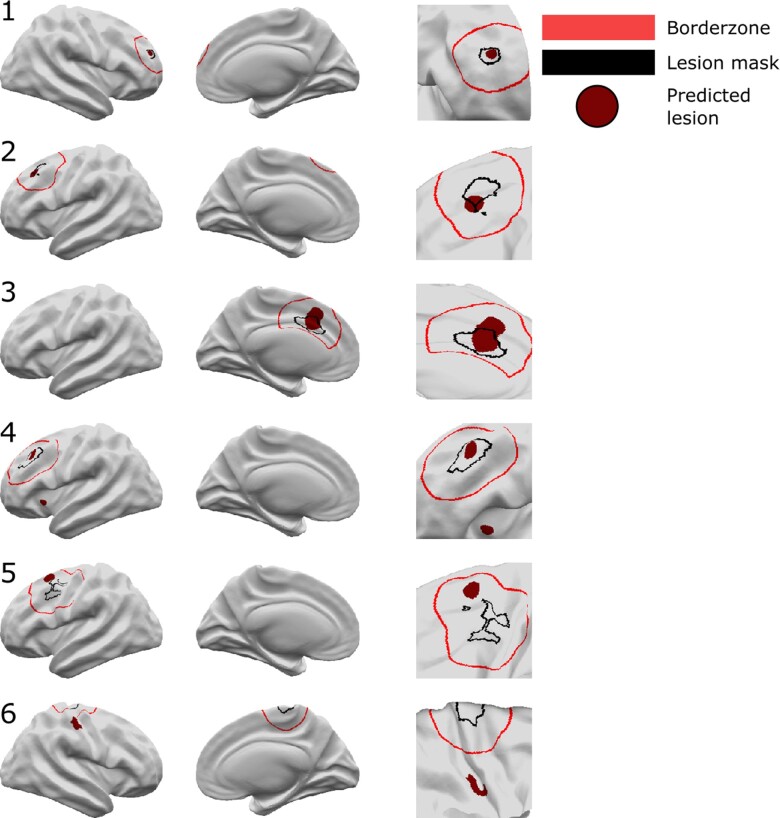
**Neural network predictions.** Classifier predictions for six patients are displayed. Patients 1–4 are examples where the classifier has correctly identified the lesion. In Patient 4 there is an additional cluster in the left insula. Patient 5 is an example where the classifier detects an area in the border zone. Patient 6 is an example of where the neural network has not identified the lesion. An additional cluster is detected in the right post-central gyrus. *Left column* = lateral view, *middle column* = medial view, *right column* = enlarged view around lesion mask. Black = lesion mask; red = border zone; burgundy = classifier-predicted clusters.

For the 260 patients in the test cohort, the classifier predicted a median of 2 (interquartile range: 1–3) clusters (Table 2). These clusters overlapped with the manual lesion mask in 154 patients (sensitivity = 59%) and overlapped with the extended lesion mask (including border zones) in 174 patients (sensitivity+ = 67%). For the 193 controls in the test cohort, the classifier predicted a median of 0 (interquartile range: 0–1) clusters. No cluster was predicted in 105/193 controls (54% specificity). Examples of individual predictions for detected and undetected lesions are presented in Fig. 3.

#### Detection in the full cohort

In the full cohort (538 patients, 373 controls), i.e. including predictions from training the network on the test dataset and testing on the train dataset, results were similar to those on the test cohort only. Sensitivity was 58%, sensitivity+ was 65% and specificity was 52% (Table 2). The classifier predicted a median of two clusters in patients and zero clusters in controls. Out of the 178 patients who were ‘ever reported MRI-negative’, clusters overlapped with the extended lesion mask (including border zones) in 112 patients (sensitivity+ = 62.9%, Table 3). On a restricted cohort of patients with T_1_ and FLAIR data, who had histopathologically confirmed FCD type IIB and were seizure-free, sensitivity was 85% (Table 3). Classifier performance according to histopathology is presented in Table 3. One hundred and thirty-five of 364 histopathologically confirmed FCDs were ‘ever reported MRI-negative’, indicating a ‘human false negative’ rate of 37%. The classifier was able to detect 69% of these challenging cases.

**Table 3:** Classifier performance grouped according to demographic factors

		% Detected	Patients (*n*)
Age group			
Adult		62.4	282
Paediatric		68.0	256
Ever-reported MRI-negative			
Visible		66.1	360
MRI negative		62.9	178
Seizure freedom			
Seizure-free		69.9	229
Not seizure-free		58.6	111
Scanner: sequence			
1.5 T: T_1_ only		46.0	63
1.5 T: T_1_ and FLAIR		82.4	34
3 T: T_1_ only		63.9	233
3 T: T_1_ and FLAIR		69.2	208
Histology			
FCD I		50.0	44
FCD IIA		64.6	113
FCD IIB		76.8	185
FCD III		72.7	22
Not available		55.7	174
Restricted cohort			
T_1_ and FLAIR, FCD IIB, seizure free		85.0	40

Detection rate per age group, MRI status, seizure freedom, scanner strengths, MRI modality, and histopathology.

#### Detection on independent test sites

When testing the classifier on the two independent sites (Table 2), sensitivity was 88% for site 1 (sensitivity+ 94%) and 56% for site 2 (sensitivity+ 62%). Specificity for site 1 was 17%, lower than expected compared to the full cohort. Performance variability is likely due to small sample sizes, which lead to large uncertainty in estimations of predictive performance.^37^ Nevertheless, these data suggest that, after data harmonization, the algorithm can generalize to detect FCDs on data from new, previously unseen sites.

### Evaluating network performance across the full cohort

#### Demographic and clinical factors affecting network sensitivity

The first logistic regression model (Supplementary Fig. 7A), based on presurgical factors, showed that lesions were more likely to be detected in patients who were operated (β = 0.43, *P* = 0.04) and those that had FLAIR data available if they were scanned on a 1.5 T MRI scanner (β = 1.10, *P* = 0.01). Lesions were less likely to be detected in patients scanned on 1.5 T scanners (β = −0.60, *P* = 0.02) and when located in the left hemisphere (β = −0.41, *P* = 0.02). However, these did not survive correction for the number of factors in the logistic regression model. There was no association with age, i.e. there was no significant difference in detection rates between paediatric and adult patients. Among post-surgical factors (Supplementary Fig. 7B), detection rates differed across histopathological subtypes, with 76.8% of FCD type IIB lesions detected, 64.6% of FCD type IIA, 72.7% in FCD type III and only 50.0% in FCD type I. FCD type I was significantly less likely (β = −0.53, *P* = 0.01) and FCD 2B more likely (β = 0.57, *P* = 0.02) to be detected than other histologies. Detection rates were non-significantly positively associated with post-surgical seizure freedom (β = 0.51, *P* = 0.04). Patients who are not seizure-free may have more subtle lesions, which may contribute to both incomplete resections and the classifier not being able to detect them. Alternatively, the lesions in patients who are not seizure-free may have been incorrectly masked.

#### MRI features of predicted lesion clusters

**Figure 4:**
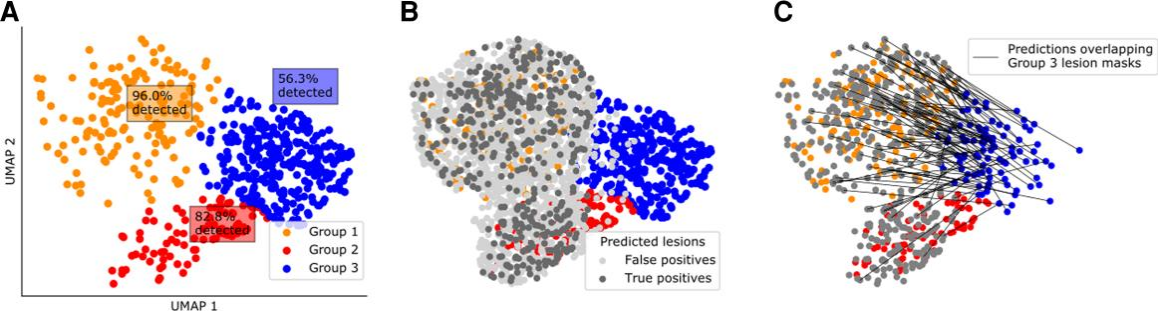
**UMAP embedding of classifier predictions.** (**A**) Data-driven clustering of UMAP embedding of lesional T_1_ features reveals three distinct groups of lesions. (**B**) True positive and false positive clusters derived from the neural network superimposed on **A**. Feature values in true positive and false positive clusters are similar to either group 1 or 2. Clusters are not similar to healthy cortex or group 3. (**C**) Predicted clusters overlapping lesion masks from group 3 lesions are superimposed. The feature values in the predicted clusters are similar to group 1 or 2, i.e. the network has identified vertices exhibiting characteristically abnormal MRI features in FCD.

The MRI features within the manually defined lesion masks clustered into three distinct groups (Fig. 4A). Groups 1 and 2 were associated with high detection rates (96.0% and 82.8%, respectively), whereas group 3, which largely overlapped healthy cortex, had much lower rates of detection (56.3%). A lower percentage of operated patients in group 3 were seizure-free (59.0% compared to 78% in groups 1 and 2). Predicted lesion clusters superimposed on this UMAP embedding entirely overlapped groups 1 and 2 (Fig. 4B) and no predicted lesion clusters were similar to group 3, which was indistinguishable from healthy cortex. For those manual lesion masks in group 3 that were correctly detected, the predicted lesion clusters exhibited features closer to those in groups 1 or 2 (Fig. 4C). This indicates that while the manual lesion masks for lesions in group 3 did not capture areas of cortical surface that exhibited characteristically abnormal MRI features, the neural network learned to identify an overlapping set of vertices that did exhibit abnormal feature characteristics.

#### Characterizing features salient to the network in segmenting focal cortical dysplasia lesions

**Figure 5:**
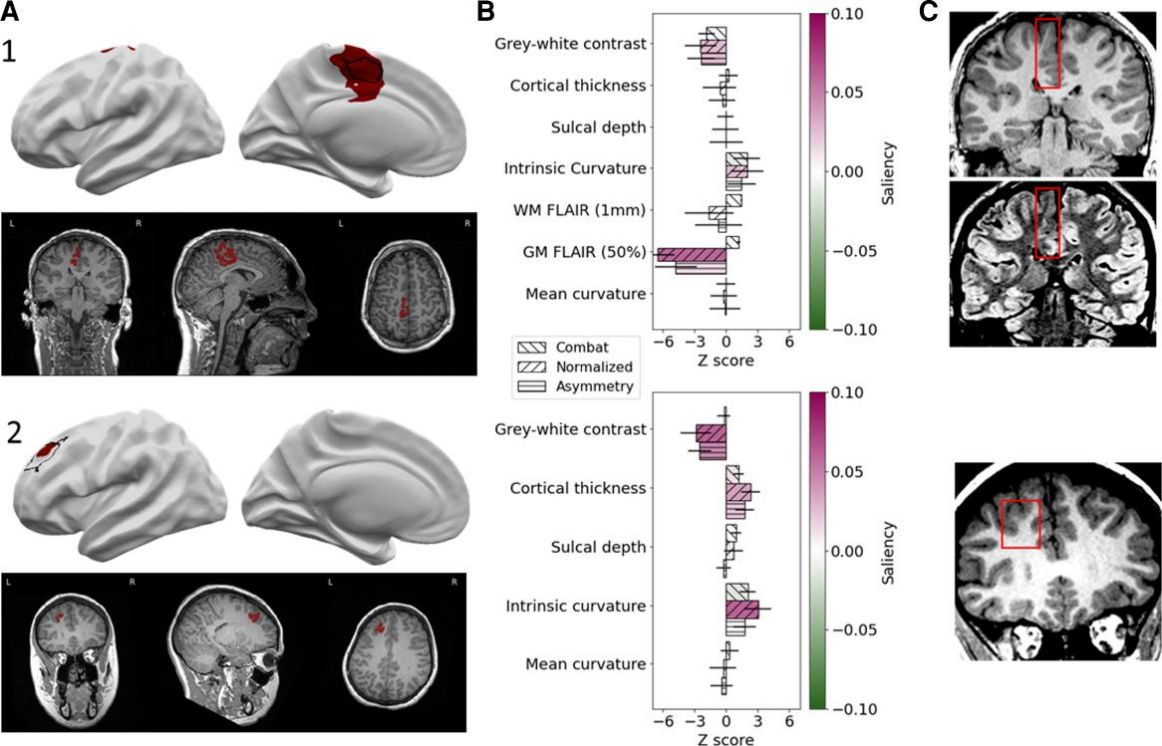
**Individual patient reports.** Example classifier predictions with saliency scores for ‘Patient 1’ (an example with FLAIR data) and ‘Patient 2’ (without FLAIR data). (**A**) Classifier predictions (dark red) and manual lesion mask (black line) visualized on brain surfaces (only lesional hemisphere is shown). Classifier predictions (dark red) visualized on T_1_ volume. (**B**) *Z*-scored mean feature values within predicted lesions coloured with Integrated Gradients saliency scores. Positive saliency scores indicate feature values driving the classifier’s ‘lesion’ prediction. Negative scores indicate feature values that are inconsistent with the prediction. (**C**) Lesional cortex highlighted on the patients’ MRI scans exhibit salient features automatically identified by the classifier.

In all patients, mean feature values and network saliencies were calculated for each feature within the predicted cluster. This enables the creation of a patient-specific report containing the predicted lesion location, which features are abnormal within that predicted cluster and how much weight those features had in driving the classifier prediction, which we illustrate in Fig. 5 with two examples. Patient 1’s predicted lesion has decreased FLAIR in the grey matter, blurring at the grey–white matter boundary on T_1_ and moderately increased intrinsic curvature (Fig. 5B). From these features, the computed saliency scores indicate that the neural network considers the decreased grey matter FLAIR and grey–white contrast most important for its prediction of lesional vertices. Patient 2 is an example of an FCD type IIB lesion without FLAIR features (Fig. 5). The predicted lesion has high intrinsic curvature, high cortical thickness and low grey–white matter boundary contrast. These are also the three features with positive saliency scores, i.e. feature values driving the classifier’s ‘lesion’ prediction.

## Discussion

We present an interpretable, fully automated pipeline for surface-based detection of FCDs, which has been validated on a large withheld test cohort, incorporating data from 20 sites, and two independent sites. The sensitivity to detect lesions in the test cohort was 67%, with sensitivities of 94% and 62% in the independent sites, 85% in subcohort with T_1_ and FLAIR data who were seizure-free with confirmed FCD IIB and 69% within patients with histologically confirmed FCD but had at some point been reported ‘MRI-negative’. Logistic regression analyses indicated that FCD type IIB lesions had higher detection rates, whereas FCD type I lesions had lower detection rates. Multidimensional analysis of lesional cortex revealed groups of lesions characterized by different MRI features, histologies, post-surgical outcomes and detection rates. Individual patient reports provide a map of the predicted lesion locations alongside the quantified lesional features and how salient they were considered by the classifier.

This study extends previous work on FCD detection in the largest MRI cohort of FCDs to date. Previous surface-based work has identified features that differentiate lesional cortex and developed machine-learning frameworks for the incorporation of these features.^14,15,17,19,38^ However, being limited by small numbers of patients and data acquired from only one or two MRI scanners can lead to large error bars on estimates of sensitivity and specificity^37^ and limited generalizability due to lack of diversity in training data. Progress is also being made on automated volumetric MRI methods.^11–13^ Both Gill *et al*.^12^ and David *et al*.^11^ report high sensitivities of 83% and 81%, respectively, on their independent test data. Within the MELD dataset, on a comparable ‘gold-standard’ subcohort of seizure-free patients with FCD type IIB who had T_1_ and FLAIR MRI data, the algorithm had a competitive sensitivity of 85%. In addition, this work differs from these studies in the following key aspects. First, this study has a more representative, heterogeneous inclusion criteria. We aimed to develop an algorithm capable of detecting all FCD histopathological subtypes including some of the more challenging FCD type I cases. Second, our classifier predicts on average two clusters per patient in our independent test sites, compared to on average six clusters per patient reported by Gill *et al*.^12^ Third, in comparison to David *et al*.,^11^ we make the code and trained model openly available, therefore fostering collaboration and clinical uptake of the work. In addition, our training dataset included lesions masked by different radiologists/researchers at different institutes. This heterogeneity in lesion masking reduced overfitting of the network to one individual neuroradiologist’s opinion. This large multisite, multiscanner cohort, including paediatric and adult data and all FCD histopathological subtypes, provided reliable and reproducible estimates of classifier performance that generalized well to two independent cohorts.

Our data-driven clustering of FCD lesions revealed three distinct groups of lesions. Group 1 had ‘classical’ radiological features of FCD type II; increased cortical thickness, blurring of the grey–white matter boundary, abnormal folding, FLAIR hyperintensity in the white matter and were often located at the bottom of sulci. They were associated with high detection rates by the neural network (96%) and had good seizure freedom rates (78%). Group 2 had more subtle features: blurring of the grey–white matter boundary, FLAIR hypointensity in the grey matter and some folding changes. However, our classifier was still able to detect 82.8% of these lesions and the patients in this group who had been operated on still had good seizure freedom rates (78%). In contrast, lesions in group 3 were difficult to differentiate from healthy cortex, they did not demonstrate characteristic FCD ‘fingerprints’ and only 59% of these patients were seizure-free after surgery. For group 3 lesions that were detected by the classifier (56.3%), the classifier identified a subset of vertices that exhibited MRI features more consistent with groups 1 and 2 (Fig. 4C). This suggests that these lesions are more subtle or difficult to delineate or structurally heterogeneous^39^ on MRI.

One challenge in incorporating machine-learning algorithms in clinical practice is their perception as being ‘black boxes’, with limited feedback on what data have informed a prediction. Saliency aims to interrogate which specific input features drive neural network predictions. Our individual patient reports provide information on which features are abnormal within the predicted clusters, accompanied by their impact on classifier prediction (Fig. 5). A neuroradiologist or multidisciplinary team could use this tool to confirm their hypotheses in ‘MRI-visible’ lesions, to re-review the scans of ‘MRI-negative’ patients or motivate more detailed investigations, such as 7 T MRI, PET or stereo EEG.^19^ They will obtain putative lesion locations identified by the classifier, equipped with an understanding of what features were considered suspicious and how they were abnormal, thus opening the ‘black box’. In addition, by ‘flagging’ suspicious areas, this artificial intelligence radiological assistant may reduce the time taken for a neuroradiologist to review MRI scans or increase confidence in the radiological diagnosis of patients with suspected FCDs.

### Limitations and future work

This study used multisite real-world data, which, while facilitating algorithm generalizability to new data and the utility of the developed tool, are heterogeneous. This heterogeneity arises from intersite differences in MRI scanners, sequences, field strengths as well as from variable post-processing operating systems and software versions and may have affected morphological and intensity feature values. These were partially mitigated through harmonization procedures but may still have impacted on algorithm sensitivity and specificity. Participating MELD sites manually masked FCD lesions and only surface-based data were shared with the project coordinators. While preserving a greater level of anonymity and facilitating data sharing, this preprocessing prevented comparison of predicted lesions with patients’ volumetric MRIs. As with other FCD detection algorithms, false positives were common in both patients and controls. This neural network classifies individual cortical vertices; future work using incorporating neighbourhood information and incorporation with volumetric approaches may help to reduce the false positives. Furthermore, volumetric approaches would extend the detection of focal epilepsy pathology beyond the neocortex, in areas such as the hippocampus. This would enable the detection of hippocampal sclerosis in FCD type IIIa. Additionally, integrating electrophysiology might help to identify which structural abnormalities are epileptogenic. One challenge in all FCD detection work is deciding which patients are considered ‘MRI-negative’. The measure ‘ever reported MRI-negative’ will vary based on the level of neuroradiological expertise at the individual site as well as the MRI scanner and sequences acquired. However, it should provide a measure of the more challenging lesions to detect. Lastly, drug-resistant focal epilepsy is caused by multiple pathologies of which FCDs are a significant subset. Invaluable future studies would extend the inclusion criteria to a wider spectrum of focal epilepsies.

## Conclusions

We demonstrate how through open-science practices and decentralized MRI post-processing, one can create a dataset; and train and validate a machine-learning framework to assist in the diagnosis of a rare, clinically challenging pathology. The MELD FCD classifier is a fully automated, open-access surface-based tool that can be run on any patient with a suspicion of having an FCD who is over the age of 3 years and has a 1.5 or 3 T T_1_ scan, with or without FLAIR data. The classifier is available on GitHub as a user-friendly Python package and can output a patient specific report detailing suspected structural abnormalities, which features are abnormal within these clusters and their impact on classifier prediction.

## Supplementary Material

awac224_Supplementary_Data

## Abbreviations

FCD: focal cortical dysplasia
FLAIR: fluid-attenuated inversion recovery
MELD: Multi-centre Epilepsy Lesion Detection
UMAP: Uniform Manifold Approximation and Projection
