# Supplementary material for: Interpretable surface-based detection of focal cortical dysplasias: a Multi-centre Epilepsy Lesion Detection study
Source: Brain. 2022 Aug 10;145(11):3859–71. doi: 10.1093/brain/awac224 (PMC9679165; doi:10.1093/brain/awac224)
Supplement: awac224_Supplementary_Data [file awac224_supplementary_data.pdf]

Supplementary Materials

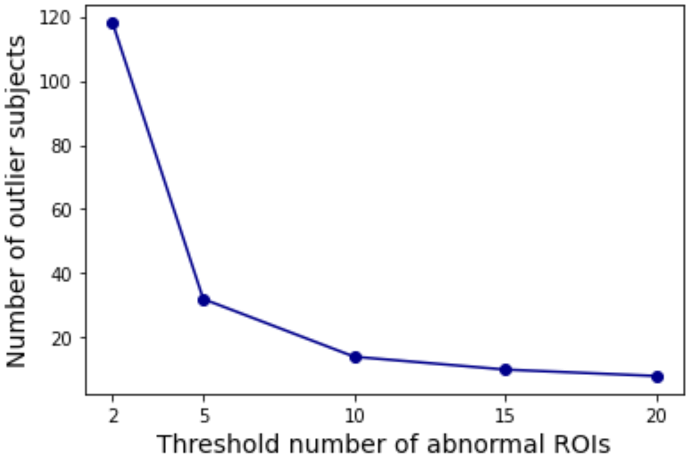

Supplementary Figure 1 Number of participants detected as outliers as the threshold number of ROIs with values of >2.7SD increases. The aim of the outlier detection was to exclude participants who likely had large systematic problems in their data, e.g. widespread FreeSurfer segmentation errors or the FLAIR co-registration misalignment, usually due to poor underlying MRI data quality. The ENIGMA Cortical Quality Control Protocol to identify abnormal ROIs (>2.7 SD). To choose the optimum number of abnormal ROIs to exclude a participant, a grid search analysis was carried out and indicated that many FCD patients had at least 2 abnormal ROIs. However, increasing the threshold to 10 ROIs, we were able to identify a small subset of subjects with widespread abnormalities indicative of extreme segmentation errors in participants' features. Increasing the outlier detection threshold to 15 or 20 abnormal ROIs only led to a small decrease of identified outlier subjects, indicating that most outlier subjects that have 10 abnormal ROIs, also have more abnormal ROIs.

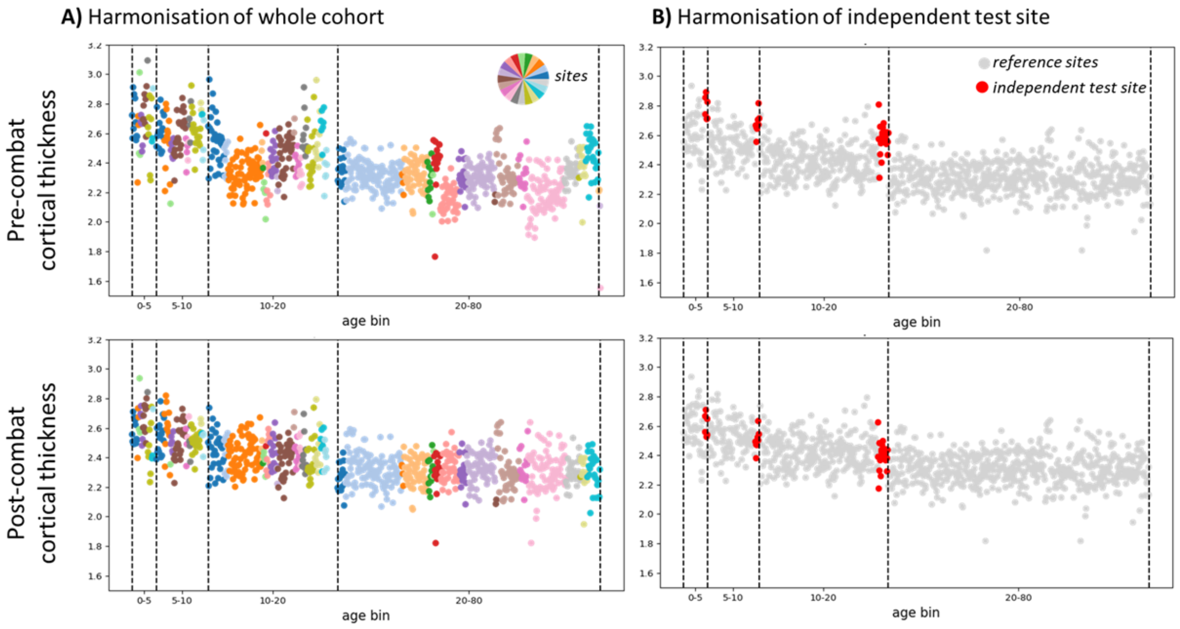

Supplementary Figure 2 Multi-centre harmonisation of cortical thickness using ComBat. (A) Pre- and post-combat cortical thickness of the whole cohort arranged per age bin and colored per site. Pre-combat site-differences

in cortical thickness are evident. Post-combat site-differences are minimised, but biological variability (e.g. age) remains - cortical thickness decreases with age. **(B)** Cortical thickness of an independent test site (red) was harmonised with the whole cohort's post-combat cortical thickness (grey) as a reference. This enables new sites to use the classifier on their MRI data.

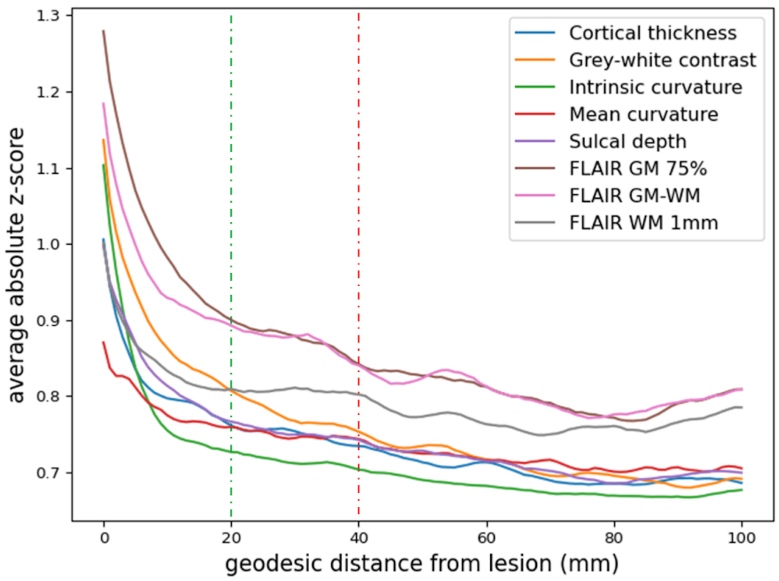

Supplementary Figure 3 Extent of lesional abnormalities beyond the manual lesion masks. Normalised feature values plotted as a function of the geodesic distance from the manual lesion mask. Abnormal feature values extend up to 40mm (red dotted line) outside the manual lesion mask. From a distance of 40mm beyond the manual lesion mask, feature values look normal (red dotted line). To reduce data and label noise, during classifier training non-lesional samples are exclusively taken from beyond a 40mm border-zone, while lesional samples are all taken from within the manual lesion masks. For more precise estimates of classifier performance, any predicted lesion cluster within the 20mm border-zone (green dotted line) is also reported in the results.

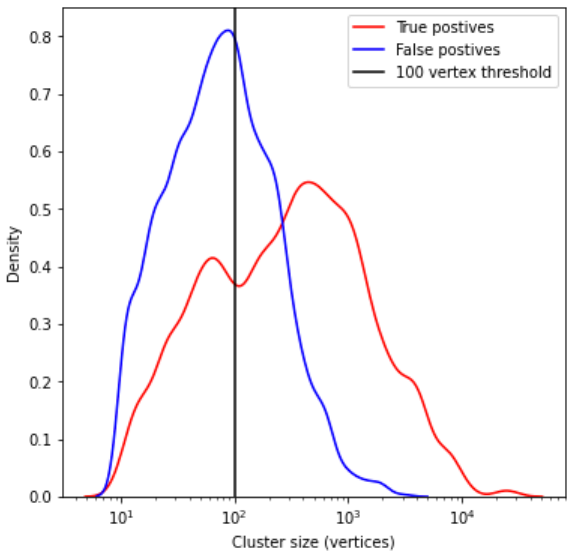

Supplementary Figure 4 Distribution of cluster sizes for false positives and true positives. True positives (red) were in general larger than false positives (blue) but their distributions overlap. Using a minimum threshold cluster size of 100 vertices (black line), eliminates the majority of false positives, while retaining most true positives.

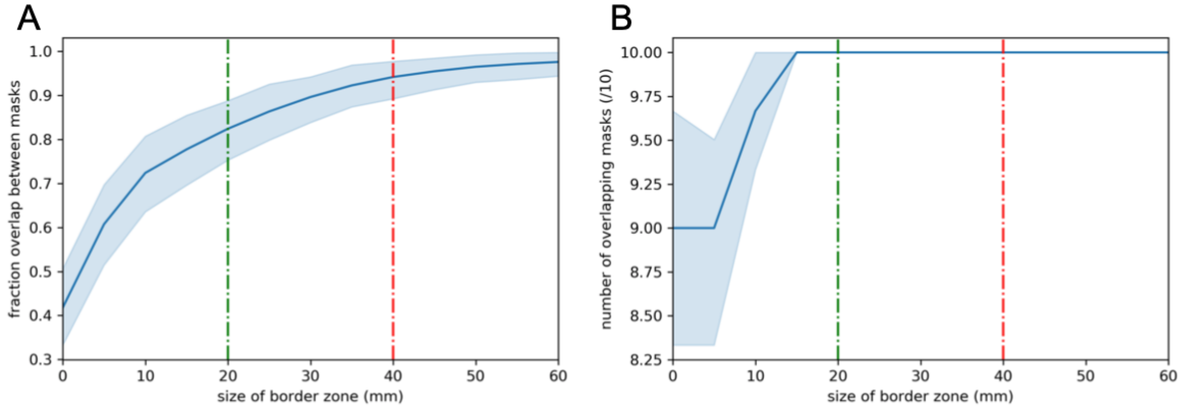

Supplementary Figure 5 Interrater agreement of three independent raters on 10 random patients from one site.

A. Mean fraction overlap between rater-rater pairs on different sizes of the border zone for the mask of the first rater. At border zones of 0mm, 20mm, 40mm, mean interrater agreement is 42%, 82%, 94%, respectively. Shaded areas show 95th confidence interval, obtained by bootstrapping.

B. Total number of overlapping masks between rater-rater pairs on different sizes of the border zone for the mask of the first rater. From a border zone of 15mm and upwards, all masks overlap in at least one vertex. Shaded areas show 95th confidence interval, obtained by bootstrapping.

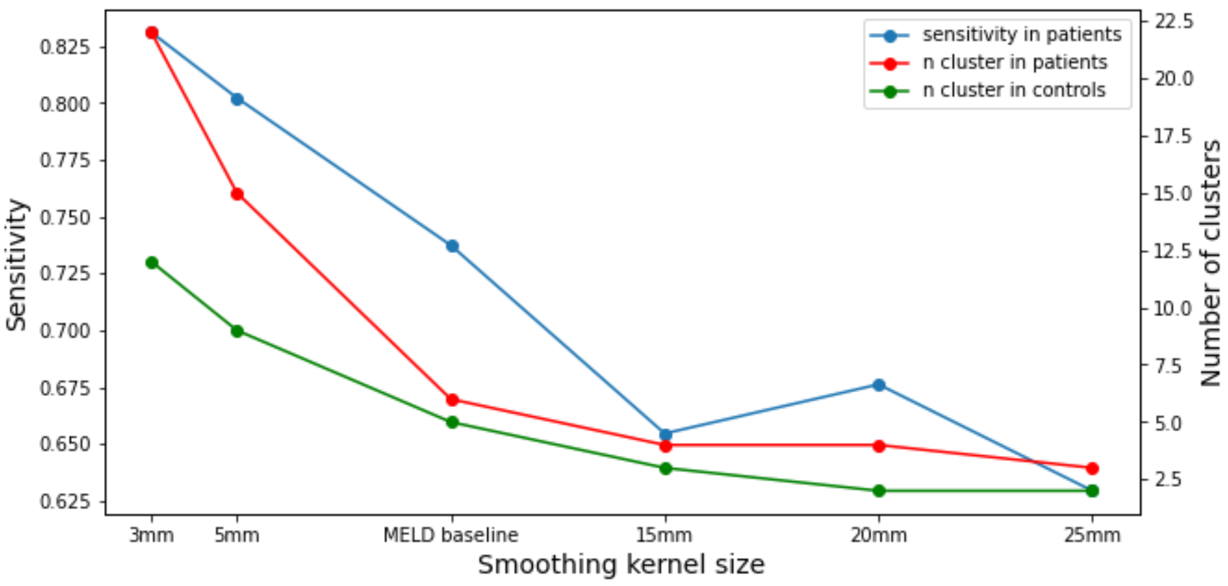

Supplementary Figure 6: Classifier sensitivity and number of clusters in the train/val cohort against smoothing kernel size (in mm) used on features. Classifier sensitivity (blue) decreases as smoothing kernel size increases. False positives (red = patients, green = controls) are extremely common with 3mm smoothing kernels, rapidly dropping off with increasing smoothing with an initial elbow at around 10mm. The experiment using the smoothing kernels used for the final training and testing of the classifier (MELD baseline) represents an acceptable trade off between falling sensitivity and rising specificity. The following Gaussian smoothing kernel sizes were used: 5mm for sulcal depth and mean curvature, 10mm for cortical thickness, grey-white contrast, and FLAIR intensities at all cortical and subcortical depths and 20mm for intrinsic curvature. These numbers reflect the sensitivity and specificity prior to a final filtering of clusters smaller than 100 vertices (Supplementary Figure 2).

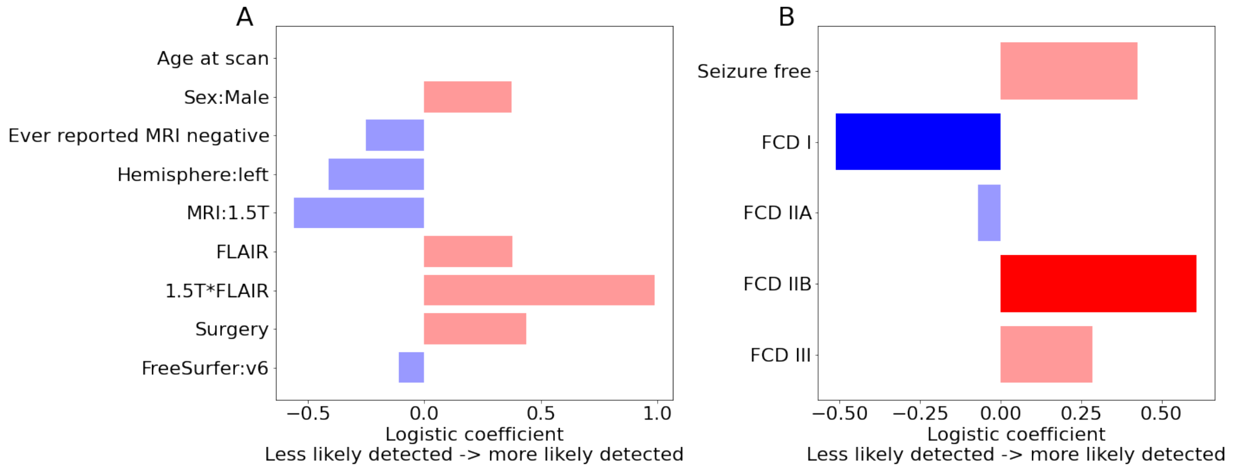

Supplementary Figure 7 Logistic regression to determine factors associated with lesion detection. A) Presurgical factors and B) post-surgical factors associated with lesion detection. Bold colours indicate significance after correction for multiple comparisons. FCD IIB lesions were significantly more likely to be detected (bold red) and FCD I lesions were significantly less likely to be detected (bold blue).

Input data selection		
Number of vertices per subject	2000 lesional, 2000 non-lesional	If there are less than 2000 lesional vertices, they are oversampled. Non-lesional vertices are drawn randomly from a pool of 10000 vertices each epoch
Size of boundary zone	40mm	Vertices from the boundary zone are excluded from training
Shuffle each epoch	True	
Normalisation	Per-feature mean and std	
Neural network architecture		
Layers	Input (xx) - Dense (40) - Dense (10) - Output (2)	
Dropout	0.4	Applied after each layer
Training		
Loss	Focal loss, alpha=0.2, gamma=5	
Learning rate	0.00001	
Batch size	1024	
Epochs	100	
Patience	10	Training was stopped early, if loss did not improve after 10 Epochs
Number of folds	10	Networks trained with 10-fold cross validation were ensembled to a final model
Evaluation		
Optimal threshold	0.52	Computed on training set:
Min area lesion	100 vertices	Minimum number of connected vertices predicted as lesion to be considered a predicted lesional cluster

Supplementary Table 1. List of neural network parameters

	**Sensitivity+** (Percentage of patients detected)	**Sensitivity** (Percentage of patients detected)	**Number of clusters in patients** (Median (IQR))	**Specificity** (Percentage of controls with zero clusters)	**Number of clusters in controls** (Median (IQR))
**Harmonised features**	54% (150/278)	44% (121/278)	3 (2 - 6)	17% (30/180)	2 (1 - 3)
**Harmonised features + asymmetry**	64% (177/278)	55% (153/278)	2 (1 - 4)	41% (74/180)	1 (0 - 2)
**Harmonised features + asymmetry + normalisation**	65% (181/278)	59% (164/180)	2 (1 - 5)	44% (79/180)	1 (0 - 2)

Supplementary Table 2 Feature normalisation improves classifier performance. Comparison of classifier performance according to feature normalisation procedures applied, using 10-fold cross-validation on the train cohort. Classifier performance is improved by successive normalisation steps, including incorporation of "asymmetry" and "normalised" features.
